# Editorial: Plant and fungal extracts and metabolites in neurotherapy: exploring their pharmacology and potential clinical uses

**DOI:** 10.3389/fphar.2025.1602574

**Published:** 2025-05-06

**Authors:** Jian Hao, Igor Lavrov, Xianhu Zhou

**Affiliations:** ^1^ Orthopedics Department, The Second Affiliated Hospital, Guangzhou Medical University, Guangzhou, China; ^2^ Department of Neurology, Mayo Clinic, Rochester, MN, United States; ^3^ Department of Physiology and Biomedical Engineering, Mayo Clinic, Rochester, MN, United States

**Keywords:** central nervous system disorders, phytochemicals, neuroprotection, ethnopharmacology, traditional medicine

Central nervous system (CNS) disorders—including Alzheimer’s disease, Parkinson’s disease, spinal cord injury (SCI), stroke, and traumatic brain injury (TBI)—continue to impose profound clinical and societal burdens (Tsai et al., 2024). Despite decades of research and significant advancements in neuroscience, most therapeutic interventions remain largely palliative, addressing symptoms rather than altering the underlying course of disease progression (Gao et al., 2020). The multifactorial and heterogeneous nature of CNS pathologies presents considerable challenges to developing effective disease-modifying therapies.

This therapeutic gap has catalyzed growing scientific interest in naturally derived bioactive metabolites, particularly phytochemicals and fungal metabolites. Long valued in traditional medical systems across diverse cultures, these metabolites are now being systematically explored for their neuroprotective, anti-inflammatory, antioxidant, and neuromodulatory properties (Koshak et al., 2017; Khazdair et al., 2019). Their multi-target pharmacological profiles, combined with favorable safety, make them compelling candidates for CNS drug discovery and development. This Research Topic features a curated Research Topic of studies that rigorously examine the therapeutic potential of plant- and fungus-derived metabolites across a range of CNS disease models, integrating ethnopharmacological knowledge with contemporary neuropharmacological approaches and methodologies.

Several studies featured in this Research Topic focus on cognitive impairment and proteinopathies within neurodegenerative contexts. Chang et al. evaluated an ethanol extract of *Rubia yunnanensis Diels (Rubiaceae)* in a rat model of chronic cerebral hypoperfusion. Their findings revealed activation of the System Xc-/GSH/GPX4 antioxidant pathway and inhibition of ferroptosis, resulting in improved cognitive outcomes (Chang et al.). Similarly, Huang et al. demonstrated that *Olea europaea L. (Oleaceae)*, a *polyphenol metabolite* extracted from olives, enhanced the proteasomal degradation of mutant huntingtin protein aggregates in Huntington’s disease cell models, independent of autophagy pathways (Huang et al.). These studies underscore the potential of phytochemicals to modulate oxidative stress and proteostasis—key processes implicated in the pathogenesis of neurodegenerative diseases.

CNS trauma, including SCI and TBI, represents another major focus within this Research Topic. Kooshki et al. reported that *Pelargonium × hortorum L.H.Bailey (Geraniaceae)*, an *anthocyanin metabolite*, significantly improved motor function and alleviated neuropathic pain in a rat model of SCI, primarily through antioxidant and anti-inflammatory mechanisms (Kooshki et al.). In a parallel study, Ma et al. developed a hydrogel-based drug delivery system for *Daphne odora Thunb. (Thymelaeaceae)*, a *coumarin metabolite* with neuroprotective activity. In a murine TBI model, this system enhanced *D. odora Thunb. (Thymelaeaceae)* bioavailability, leading to improved spatial memory and reduced neuroinflammation (Ma et al.). These studies highlight the value of innovative formulation strategies in optimizing the efficacy of natural metabolites for acute CNS injuries.

Additional insights into SCI therapeutics are provided by Hao et al. (2019) who investigated the neuroprotective effects of rhein lysinate (RHL) (Hao et al., 2019). In SCI rats, RHL administration significantly improved motor function, increased the activity of endogenous antioxidant enzymes (SOD and GSH-Px), and reduced lipid peroxidation, as indicated by lower MDA levels. Mechanistically, RHL inhibited activation of the p38 MAPK signaling pathway, thereby attenuating neuronal apoptosis. These findings suggest that RHL promotes neuroprotection through the modulation of oxidative stress and apoptotic pathways.

In a subsequent study, the same group demonstrated that *Syringa vulgaris L. (Oleaceae)*, a *lignan metabolite*, facilitates functional recovery following SCI by activating the UBE4B/AKT signaling pathway. In glutamate-induced neurotoxicity models using SH-SY5Y cells, *S. vulgaris L. (Oleaceae)* upregulated UBE4B expression and enhanced AKT phosphorylation, resulting in reduced neuronal apoptosis. The protective effects were abolished by either UBE4B knockdown or AKT inhibition, confirming the critical role of this signaling axis (Hao et al., 2024). These findings reinforce the therapeutic relevance of the UBE4B/AKT pathway and underscore the broader potential of lignan metabolites in CNS repair and regeneration.

Pain management, particularly for chronic and neuropathic pain, is another area where phytotherapeutics offer considerable promise. Wang et al. showed that *Zingiber officinale Roscoe (Zingiberaceae)*, a *bioactive metabolite* derived from Zingiber officinale (ginger), mitigated inflammatory pain by suppressing neuronal excitability in the anterior cingulate cortex, suggesting a central mechanism of analgesia (Wang et al.). Complementarily, Hashemi et al. reported that *Haematococcus pluvialis Flotow (Haematococcaceae)*, a marine *carotenoid metabolite*, alleviated neuropathic pain in a chronic constriction injury model. Its mechanism of action involved both oxidative stress reduction and modulation of opioid and benzodiazepine receptor pathways, reflecting its multi-target pharmacological properties (Hashemi et al.).

Age-related cognitive decline and neuronal senescence were explored by Garg et al., who investigated *Glycine* max *(L.) Merr. (Fabaceae)*, a soybean-derived oligopeptide. In a doxorubicin-induced hippocampal aging model, *Glycine* max *(L.) Merr. (Fabaceae)* activated the Wnt/β-catenin signaling pathway, suppressed senescence markers such as p16^INK4a and p21, and improved memory performance (Garg et al.). Similarly, Wu et al. assessed the effects of *Glycyrrhiza uralensis Fisch. ex DC. (Fabaceae)* in a neonatal white matter injury model. The *metabolite* promoted oligodendrocyte maturation and reduced microglial activation through inhibition of histone deacetylase 3 (HDAC3), suggesting that phytochemicals may exert neuroprotective effects via epigenetic modulation (Wu et al.).

Sleep and mood disturbances, commonly observed in neurodegenerative and psychiatric disorders, were addressed by Li et al. using *Eucalyptus globulus Labill. (Myrtaceae)* (EEO) (Li et al.). Their study demonstrated that EEO increased the levels of inhibitory neurotransmitters, including GABA and glycine, and modulated the composition of gut microbiota. These findings support the hypothesis that EEO exerts its sedative-hypnotic effects through gut–brain axis interactions, offering a novel mechanistic perspective on plant-based interventions for sleep disorders.

Clinical evaluations of traditional *botanical drug* formulations were also featured. Chang et al. conducted a meta-analysis on the use of *Sophora flavescens Aiton (Fabaceae)* in patients experiencing bone neuropathic pain secondary to tumor metastasis. The analysis revealed significant reductions in pain severity and improvements in functional outcomes, validating its therapeutic potential in oncology-related pain management (Chang et al.). Jiao et al. investigated the Yulinzhu botanical drug formula in women with polycystic ovary syndrome (PCOS), a condition involving neuroendocrine dysregulation (Jiao et al.). Their findings demonstrated improvements in ovulation rates and hormonal profiles, indicating potential modulation of the hypothalamic–pituitary–gonadal axis. These results emphasize the systemic therapeutic potential of CNS-targeted phytomedicine in broader physiological contexts.

Collectively, the contributions in this volume offer a comprehensive overview of recent progress in plant- and fungus-derived metabolites for CNS disorders. These studies address a wide spectrum of conditions—from neurodegeneration and trauma to chronic pain, neurodevelopmental injury, and neuroendocrine dysfunction—highlighting the ability of natural metabolites to influence key pathological processes such as oxidative stress, inflammation, apoptosis, proteostasis imbalance, and epigenetic dysregulation.

Critically, while the studies in this volume have advanced our understanding of the therapeutic potential of plant- and fungus-derived metabolites, several limitations remain. Many investigations rely on preclinical models that do not fully capture the complexity of human CNS disorders, and standardized methodologies for the development of botanical drugs are still lacking. Moreover, the pharmacokinetic profiles and long-term safety of several metabolites have not been comprehensively evaluated.

Future research should therefore prioritize the design and execution of robust clinical trials to validate these preclinical findings. Additionally, more in-depth mechanistic studies are needed to elucidate the molecular targets of these metabolites, and efforts should be made to optimize extraction, purification, and standardization protocols for botanical drugs. Interdisciplinary approaches that integrate ethnopharmacology, neurobiology, and advanced drug delivery systems will be crucial to translating these promising findings into effective therapeutic interventions. Such research topics will not only help bridge the gap between preclinical promise and clinical application but will also set the scene for further developments in CNS pharmacotherapy.
